# Heart-brain axis: low blood pressure during off-pump CABG surgery is associated with postoperative heart failure

**DOI:** 10.1186/s40779-024-00522-x

**Published:** 2024-03-20

**Authors:** Xiu-Yun Liu, Jing-Jing Mu, Jian-Ge Han, Mei-Jun Pang, Kuo Zhang, Wen-Qian Zhai, Nan Su, Guang-Jian Ni, Zhi-Gang Guo, Dong Ming

**Affiliations:** 1 Academy of Medical Engineering and Translational Medicine, Tianjin University, Tianjin, 300072 China; 2 State Key Laboratory of Advanced Medical Materials and Devices, Tianjin, 300072 China; 3 Haihe Laboratory of Brain -Computer Interaction and Human-Machine Integration, Tianjin, 300380 China; 4 Department of Anesthesiology, Tianjin University Chest Hospital, Tianjin Key Laboratory of Cardiovascular Emergency and Critical Care, Tianjin Municipal Science and Technology Bureau, Tianjin, 300222 China; 5 Department of Cardiac Surgery, Tianjin University Chest Hospital, Tianjin Key Laboratory of Cardiovascular Emergency and Critical Care, Tianjin Municipal Science and Technology Bureau, Tianjin, 300222 China

**Keywords:** Off-pump coronary artery bypass grafting (CABG), Heart failure (HF), Individualized arterial blood pressure (ABP) management, Cerebral autoregulation (CA), Optimal ABP

Dear Editor,

The primary objective of the letter is to emphasize the importance of personalized management of arterial blood pressure (ABP) in the context of off-pump coronary artery bypass grafting (CABG) surgery. Coronary artery disease, a leading global cause of mortality, necessitates a substantial number of cardiac surgeries, with approximately 400,000 CABG operations conducted annually in the United States. Postoperative heart failure (HF) is a common occurrence after CABG surgery, with readmission rates within 30 d due to HF ranging from 12 to 16%. Researchers have highlighted the critical role of HF management before, during and after CABG surgery, identifying hemodynamic instability, perioperative myocardial injury, and low cardiac output syndrome as predictive factors for postoperative HF. In 2023, Han et al. [1] found that factors such as pulse index failure and composite grafting are independent predictors of CABG failure. Additionally, a study reported by Loncar et al. [2] revealed a significant relationship between reduced cerebral blood flow (CBF) and the severity of HF in elderly males. Moreover, Hartono et al. [3] pointed out that perioperative myocardial injury and pre-existing left ventricular systolic dysfunction can contribute to post-CABG HF. In the surgical setting, the reduction of mean arterial pressure (MAP) to minimize collateral bleeding and enhance surgical visualization often leads to low systemic perfusion. Currently, the prevailing approach in CABG surgeries involves a uniform MAP management strategy, typically targeting a range of 60 to 70 mmHg for most patients. However, given the varying clinical backgrounds of patients and their diverse tolerance to low MAP levels, there is an urgent need for personalized MAP management during CABG surgery to ensure adequate blood flow and prevent postoperative complications.

The brain maintains stable perfusion through cerebral autoregulation (CA), which allows it to regulate CBF despite changes in ABP. Rhee et al. [4] have previously demonstrated that under severe hypoperfusion, the body may prioritize protecting the brain over peripheral organs such as the kidneys. By inducing continuous blood loss in piglets, they observed a reduction in kidney blood flow preceding a decrease in CBF. Previous studies by our researchers and others have established a correlation between low blood pressure and reduced cerebral perfusion during CABG and postoperative complications, including delirium, acute kidney injury, major morbidity, and operative mortality [5–7]. However, the specific MAP target most strongly associated with HF remains unknown. Therefore, the hypothesis of our current study is that low MAP and reduced cerebral perfusion to the brain might be a strong indicator of systemic ischemia and HF.

Various parameters have been developed to monitor intraoperative CA in real-time, including cerebral oxygen saturation index (COx), which is determined by the correlation between MAP and regional cortical oxygen saturation (rSO_2_). COx further facilitates the promotion of personalized MAP management guideline by identifying specific MAP targets, such as the lower limit of autoregulation (LLA), the upper limit of autoregulation (ULA), and optimal mean arterial pressure (MAPopt). In the past several decades, these targets have been examined to prevent postoperative complications after on-pump CABG, but concerns have been raised about the reliability of these findings due to the dampening of blood pressure fluctuations during pump-driven circulation. As pointed out by Claassen et al. [8] and Gelpi et al. [9], the natural variability of MAP is extremely important in CA, emphasizing the dynamic relationship between blood pressure and CBF as a high-pass filter that may be attributed to the slow adaptation (< 0.2 Hz) of cerebral arterioles in response to fast fluctuations of perfusion pressure (< 0.2 Hz). Consequently, there is growing interest in off-pump CABG surgery due to the potential benefits of reduced stroke risk. Further investigation is warranted to establish personalized MAP management for off-pump CABG patients.

In this study, we have introduced a personalized approach to managing MAP during off-pump CABG surgery to address the aforementioned challenges and the whole process is shown in Fig. 1. Utilizing a sampling frequency of 1 Hz, ABP and brain oxygen saturation (rSO_2_) were concurrently monitored in a group of patients undergoing off-pump CABG at Tianjin Chest Hospital (Tianjin, China), as shown in Additional file 1: Fig. S1. Individualized MAPopt, LLA, and ULA were determined by calculating COx for each patient. A previously established multi-window curve-fitting algorithm was used to construct a “U-shaped” curve (Additional file 1: Fig. S2) [10]. The relationship between the extent of MAP below a certain threshold and patient outcome was assessed by computing area under the curve (AUC) of MAP below MAPopt (or LLA). The AUC for MAP exceed in ULA was also calculated. Additionally, the percentage of time spent with MAP below MAPopt (or LLA) and above ULA was analyzed. Data integration was performed using Matlab software and subsequent analysis was conducted using the software of intensive care monitoring plus (ICM +). For statistics, we used a logistic regression model adjusted for age, diabetes, aspartate aminotransferase/alanine transaminase (AST/ALT) and log EuroSCORE (determined prior to possible confounding variables). For more details, please refer to Additional file 1: Material and methods.

**Fig. 1:**
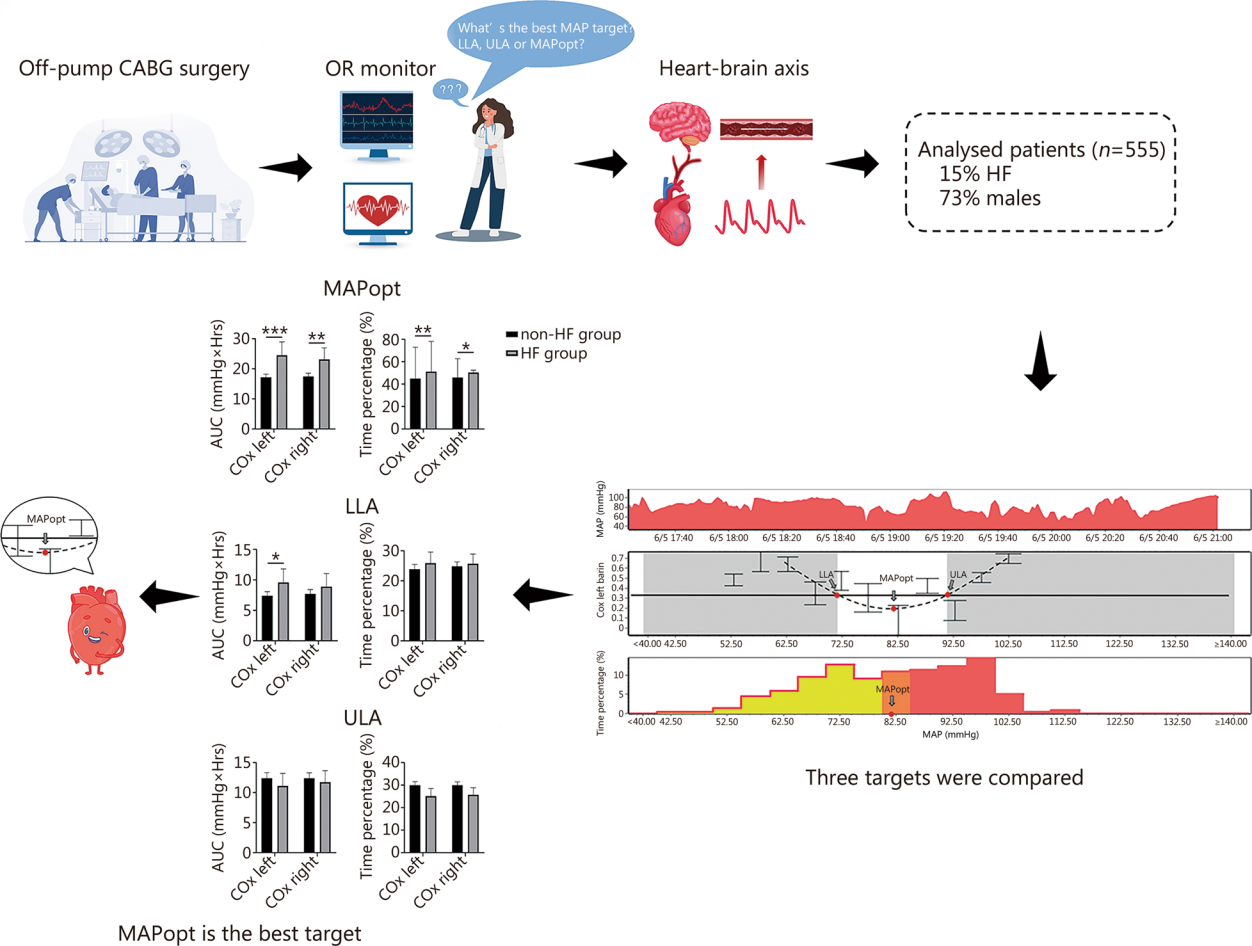
Personalized arterial blood pressure (ABP) management potentially reduces the risk of heart failure (HF) following off-pump coronary artery bypass grafting (CABG). Clinicians in the operating room may face uncertainty regarding the optimal target ABP level to reduce postoperative complications. This research involved monitoring ABP and brain oxygen saturation concurrently in a group of patients undergoing off-pump CABG surgery. Through assessment of continuous cerebral autoregulation, three specifics targets, namely lower limit of autoregulation (LLA), upper limit of autoregulation (ULA), and optimal mean arterial pressure (MAPopt), were identified. The findings indicated a significant association between HF and the degree of ABP falling below the optimal range. COx cerebral oxygen saturation index, AUC area under the curve

The data from a total of 555 patients were analyzed and divided into two groups based on the presence or absence of HF, with 85 patients in the HF group and 470 patients in the non-HF group. The mean age of the cohort was 66 years (ranging from 39 to 84 years), with 73.3% male (Additional file 1: Fig. S3, Table S1). The key findings can be summarized as follows. (1) In both the unadjusted model and the model adjusted by age, diabetes, AST/ALT, and log EuroSCORE, a significant association was observed between the degree of MAP falling below the MAPopt and the occurrence of HF after off-pump CABG. Patients in the HF group exhibited higher AUC [24.5 (20.1–29.0) vs. 17.2 (16.1–18.2), *P* < 0.001] and longer durations of low MAP [(51.1 ± 27.3)% vs. (44.9 ± 28.2)%, *P* = 0.002] compared to those in the non-HF group (Additional file 1: Fig. S4, Table S2). However, there was no significant correlation observed between MAP < LLA and MAP > ULA with patient outcomes. (2) Compared to the non-HF group, patients in the HF group tended to exhibit higher variability in MAP (standard deviation, *P* = 0.046) and heart rate variability (*P* = 0.001), indicating a close relationship between hemodynamic instability and HF, as also noted by Hartono et al. [3]. (3) Furthermore, the study revealed that patients with HF tended to have lower intraoperative rSO_2_ levels and reduced left ventricular ejection fraction before surgery. The degree of MAP falling below MAPopt (i.e., MAPopt-MAP) showed a negative relation with cardiac output or stroke volume (Additional file 1: Table S2, Fig. S5), suggesting that reduced cerebral perfusion is closely associated with decreased cardiac output and stroke volume.

This research underscores the significance of personalized blood pressure management in off-pump CABG surgery, with MAPopt identified as the optimal target to reduce the incidence of postoperative HF. Patients who experience longer duration and larger extent of low blood pressure are at an elevated risk of developing HF after CABG. Therefore, the implementation of a personalized MAP management strategy guided by MAPopt should be considered for off-pump CABG surgery in forthcoming clinical practice.

### Supplementary Information

**Additional file 1:** Material and Methods. **Fig. S1** Real-time recording of intraoperative signals. **Fig. S2** Personalized MAPopt, LLA, and ULA defined a multi-window curve-fitting algorithm. **Fig. S3** Patient flowchart. **Fig. S4** Comparison of AUC and time percentage between patients based on the three ABP targets. **Fig. S5** Relationship between MAPopt-MAP and cardiac output or stroke volume. **Table S1** Patient details. **Table S2** The association between the tested parameters and heart failure outcome.

## Data Availability

The datasets used and analyzed during the current study are available from the corresponding author (xiuyun_liu@tju.edu.cn) on reasonable request.

## Abbreviations

ABP: Arterial blood pressure
AUC: Area under the curve
CA: Cerebral autoregulation
CABG: Coronary artery bypass grafting
CBF: Cerebral blood flow
COx: Cerebral oxygen saturation index
LLA: Lower limit of autoregulation
MAP: Mean arterial pressure
MAPopt: Optimal mean arterial pressure
rSO_2_: Regional cortical oxygen saturation
ULA: Upper limit of autoregulation
