# Supplementary material for: Heart-brain axis: low blood pressure during off-pump CABG surgery is associated with postoperative heart failure
Source: Mil Med Res. 2024 Mar 20;11:18. doi: 10.1186/s40779-024-00522-x (PMC10956228; doi:10.1186/s40779-024-00522-x)
Supplement: Supplementary file 1 — Additional file 1: Material and Methods. Fig. S1 Real-time recording of intraoperative signals. Fig. S2 Personalized MAPopt, LLA, and ULA defined a multi-window curve-fitting algorithm. Fig. S3 Patient flowchart. Fig. S4 Comparison of AUC and time percentage between patients based on the three ABP targets. Fig. S5 Relationship between MAPopt-MAP and cardiac output or stroke volume. Table S1 Patient details. Table S2 The association between the tested parameters and heart failure outcome. [file 40779_2024_522_MOESM1_ESM.pdf]

Material and methods

The study was approved by the ethical committee of Tianjin Chest Hospital (Tianjin, China) (2020YS-022-01). A written consent form was obtained from each patient or the next of kin.

Patients

Patients undergoing off-pump coronary artery bypass grafting (CABG) were enrolled between June 8, 2021, and February 24, 2022, at Tianjin Chest Hospital. The recruitment criteria include age > 18 years old, undergoing isolated or combined cardiac artery bypass graft, valve, aortic, or myomectomy surgery. Patients were excluded if they got a lung or heart transplant, insertion of a ventricular assist device, or baseline dialysis. The exclusion criteria are too short or duplicated records during surgery, or no outcome records.

Data collection

Regional cortical oxygen saturation (rSO_2) was monitored through two near infrared spectroscopy (NIRS) probes by NONIN (Nonin Medical Inc., Plymouth, USA) placed on the patient's forehead. Respiratory and vital signals were collected by PHILIPS (IntelliVue MX500, Royal Dutch Philips Electronics Ltd., Amsterdam, The Netherlands) or GE monitor (Dash 4000, General Electric Company, Boston, USA). Cardiac output (CO) and heart rate (HR) were recorded by LiDCO (Masimo company's LiDCO Haemodynamic Monitoring System, London, UK). Arterial blood pressure (ABP) was monitored invasively via a radial artery. All data were recorded continuously with a sampling frequency of 1 Hz during the whole surgery in the operating room, as shown in **Additional file 1: Fig. S1**. Data integration was performed using Matlab software (ver. R2019b, MathWorks, Inc., Massachusetts, USA) and

subsequent analysis was conducted using the software of intensive care monitoring plus (ICM+) (University of Cambridge, Cambridge Enterprise, Cambridge, UK, https://icmplus.neurosurg.cam.ac.uk). Artefacts introduced by tracheal suctioning, arterial line flushing, or transducer malfunction were removed by ICM+.

Perioperative care

Perioperative care was based on usual clinical practice. General anesthesia was induced and maintained with 0.03 – 0.05 mg/kg midazolam, 0.15 – 0.3 mg/kg etomidate, 0.5 – 1.0 μg/kg sufentanil, and 0.6 mg/kg rocuronium for all the enrolled patients. Identical general anesthetics, analgesics, and muscle relaxants were performed during anesthesia maintenance, i.e., propofol, sevoflurane, and sufentanil were used. The partial pressure of carbon dioxide was kept between 35 to 45 mmHg. The rewarming goal is pharyngeal temperature < 37 °C based on institutional standards. During off-pump CABG, MAP goals are established based on discussions among surgeons, anesthesiologists, and perfusionists, and usually, MAP is kept above 65 mmHg. The goal in the ICU was 65 – 90 mmHg, inotropes were based on the estimation of adequate perfusion. After surgery, the patient was transferred to the ward.

Defining personalized MAP target

As mentioned above, the NIRS-based COx was calculated as the moving Pearson correlation coefficient between the 10-second mean of MAP and rSO_2, using a 300-second window. A negative or near-zero COx value refers to functional autoregulation, indicating stable CBF despite fluctuations in MAP [1]. A COx value close to one means impaired autoregulation, showing a close relationship between CBF and MAP. A previously established multi-window curve-fitting algorithm was used to construct a "U-shaped" curve [2] (**Additional file 1: Fig. S2)**. Firstly, COx was assembled and placed into 5 mmHg MAP bins. The

MAP range for MAPopt calculation was 40 to 120 mmHg. Then, for each MAP bin, the mean value and standard error of COx were calculated and plotted against the mean MAP to create the error bar chart, representing the relationship between COx and MAP. Then, the multi-window curve-fitting algorithm was used to the error bar to form a "U-shaped" curve, with the best CA at the lowest COx point [3, 4]. The associated MAP value was defined as the MAPopt. The LLA and ULA were determined by drawing a straight horizontal line at the threshold of COx = 0.35 [5]. The x-coordinate of the point where the horizontal line intersects the "U-shaped" curve on the left side [6, 7] was defined as LLA, and the intersection point on the right side was defined as ULA, as shown in **Additional file 1: Fig. S2**.

AUC of MAP below MAPopt (or LLA) and the AUC of MAP above ULA

To quantify the relationship between extension of MAP below a certain threshold and patient outcome, we calculated the AUC of MAP below MAPopt (or LLA) using the following formula:

$$\sum_{i=0}^{n}(\text{Magnitude i} \times \Delta\text{Time}) \ \ [\text{mmHg} \times \text{h}],$$

where magnitude (mmHg) means the extension of MAP deviation below MAPopt (or LLA) and time (hours) is the duration of MAP below MAPopt (or LLA). The AUC of MAP above ULA was also calculated using the above formula, where the magnitude refers to the extension of MAP above ULA, and the time means the duration of MAP above ULA. Meanwhile, the time percentage of MAP below MAPopt (or LLA) and the time percentage of MAP above ULA were also calculated as the ratio of the time duration of the related event to the whole recording time.

Heart failure (HF)

The endpoint of this study is the occurrence of postoperative HF. The 2021 European Society of Cardiology guidelines define the diagnosis and treatment of HF [8]. According to the guidelines, the

diagnosis of HF is based on the symptoms, physical examination, electrocardiogram, brain natriuretic peptide (BNP) level, or N-terminal BNP precursor (NT proBNP) plasma concentration, chest radiograph examination, echocardiography, and other imaging of patients by cardiologists.

Statistical analysis

Statistical analysis was conducted using SPSS (version 24.0, IBM, NY, USA). The patients were divided into two groups: HF group and non-HF group. The mean value of AUC and time percentage of MAP < MAPopt (or LLA) or MAP > ULA were calculated for each patient during the whole surgery, and were compared between the two patient groups using logistic regression models adjusted for age, diabetes mellitus, aspartate aminotransferase/alanine transaminase (AST/ALT), and log EuroSCORE (determined a priori for possible confounding variables). For all analyses, *t*-tests were used for normal distributions and the data were shown by mean ± SD, nonparametric tests were used for non-normal distributions and 95%CI was applied, with *P*-value < 0.05 considered statistically significant.

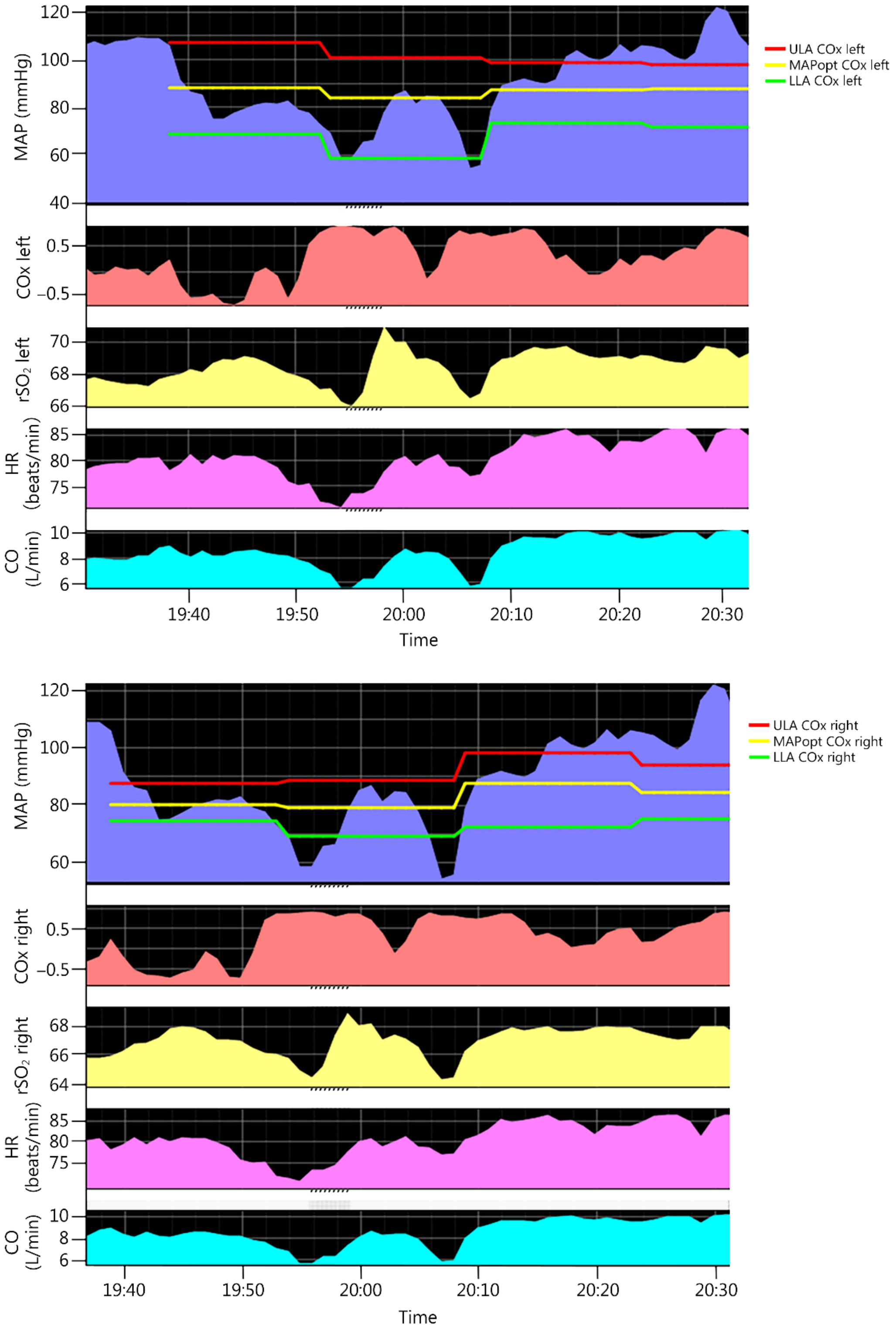

Fig. S1 Real-time recording of intraoperative signals. ULA upper limit of autoregulation, MAPopt optimal mean arterial pressure, LLA lower limit of autoregulation, MAP mean artery pressure, COx cerebral oximetry index, COx left COx value based on left brain rSO_2, COx right COx value based on right brain rSO_2, rSO_2 regional cerebral oxygen saturation,

HR heart rate, CO cardiac output, rSO_2 left regional cerebral oxygen saturation based on left brain, rSO_2 right regional cerebral oxygen saturation based on right brain

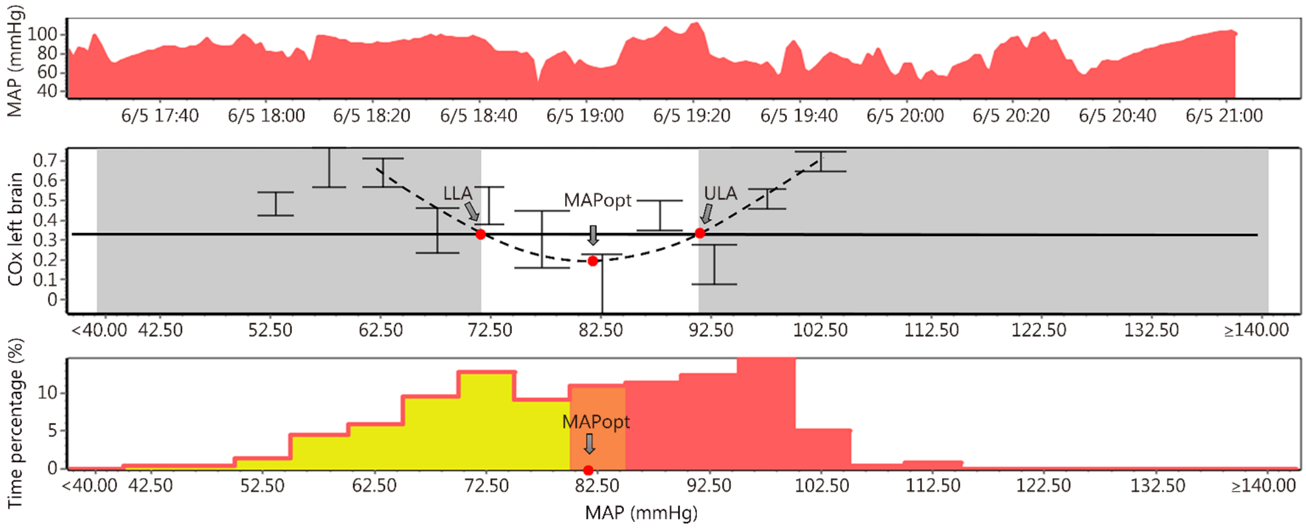

Fig. S2 Personalized MAPopt, LLA, and ULA defined a multi-window curve-fitting algorithm. The MAP at the turning point of the "U-shape" curve in the middle panel is defined as MAPopt. The left point where the horizontal line of COx = 0.35 intersects the U-shaped curve is defined as LLA. The right point where the horizontal line of COx = 0.35 intersects the "U-shaped" curve is defined as ULA. The bottom panel shows the area under the curve of MAP < MAPopt. MAP mean artery pressure, MAPopt optimal mean artery pressure, COx left left side cerebral oximetry index, LLA lower limit of autoregulation, ULA upper limit of autoregulation

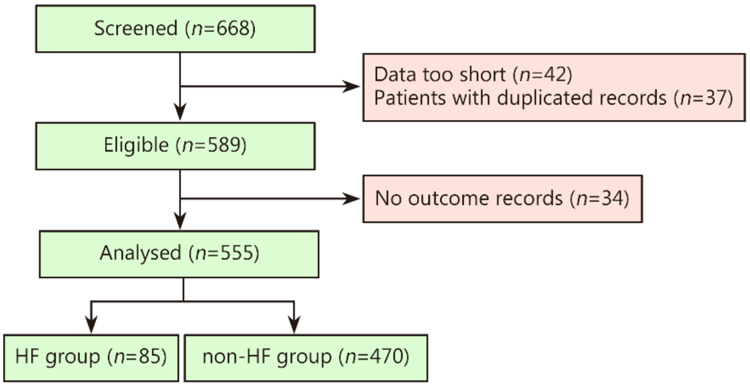

Fig. S3 Patient flow chart. HF heart failure

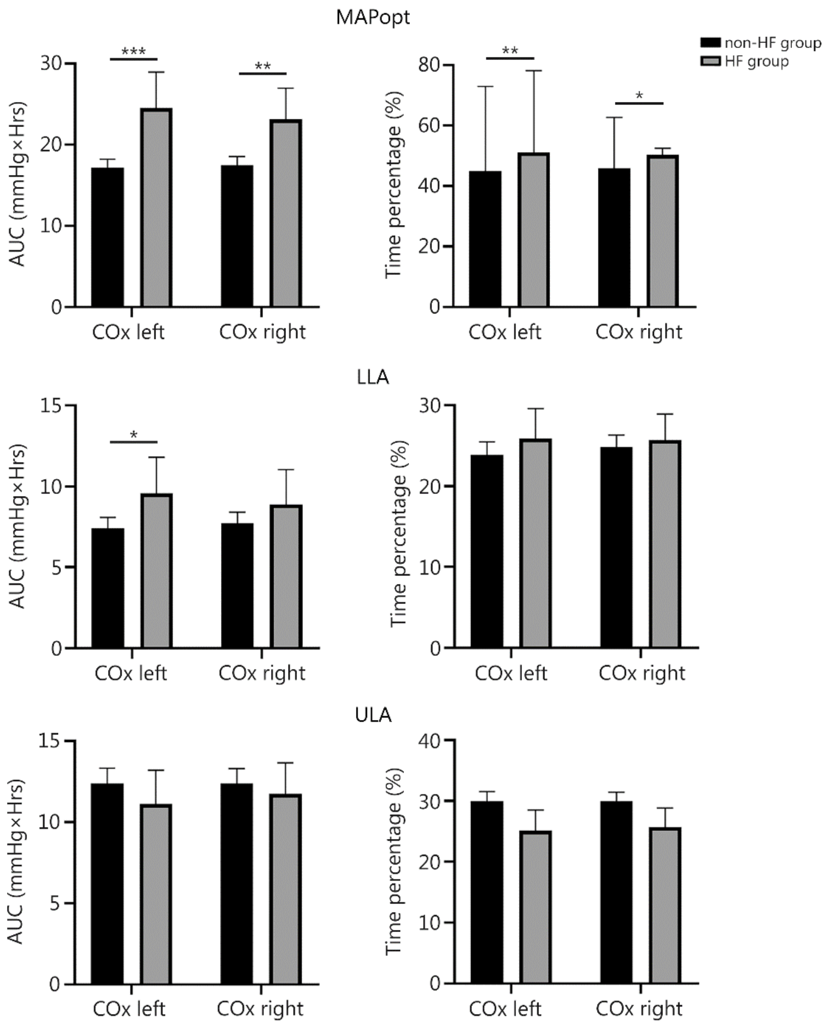

Fig. S4 Comparison of AUC and time percentage between patients based on the three ABP targets. AUC area under curve, MAPopt optimal mean arterial pressure, LLA lower limit of autoregulation, ULA upper limit of autoregulation, COx left COx value based on left brain regional cerebral oxygen saturation, COx right, COx value based on right brain regional cerebral oxygen saturation, HF heart failure. Level of significance was annotated as $^{*}P < 0.05$, $^{**}P < 0.01$, $^{***}P < 0.001$

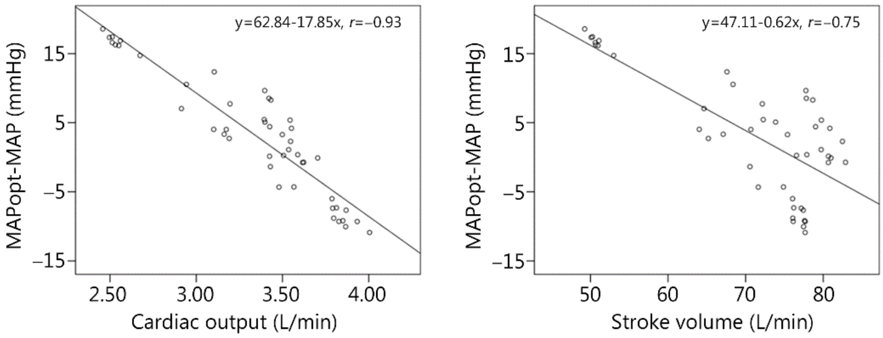

Fig. S5 Relationship between MAPopt-MAP and cardiac output or stroke volume. MAPopt optimal mean arterial blood pressure

Table S1 Patient details (*n* = 555)

Characteristic	Values
MAP (mean ± SD)	76.8 ± 8.1
COx left (mean ± SD)	0.3 ± 0.1
COx right (mean ± SD)	0.3 ± 0.1
MAPopt COx left (mean ± SD)	74.5 ± 8.7
MAPopt COx right (mean ± SD)	74.7 ± 8.6
CO (L/min, mean ± SD)	5.0 ± 1.4
LLA COx left (mean ± SD)	66.5 ± 9.1
LLA COx right (mean ± SD)	66.9 ± 9.1
ULA COx left (mean ± SD)	82.8 ± 9.7
ULA COx right (mean ± SD)	82.7 ± 10.1
Male sex [*n* (%)]	407 (73.3)
Age [years, median (range)]	66 (39 – 84)
Height (cm, mean ± SD)	168.4 ± 7.5
Weight (kg, mean ± SD)	73.9 ± 25.9
BMI (mean ± SD)	26.0 ± 8.8
Current smoker [*n* (%)]	326 (58.7)
Current drinker [*n* (%)]	134 (24.1)
Prior DM [*n* (%)]	235 (42.3)
Prior hypertension [*n* (%)]	385 (69.4)
Mean EuroSCORE [median (IQR)]	3.4 (2.0 – 5.0)
Prior stroke [*n* (%)]	6 (1.1)
Duration of ICU (h, mean ± SD)	2.2 ± 0.9

MAP mean artery pressure, *COx left* cerebral oximetry index based on left brain rSO_2, *COx right* cerebral oximetry index based on right brain rSO_2, rSO_2 regional cerebral oxygen saturation, *MAPopt COx left* optimal mean arterial pressure calculated with left brain Cox, *MAPopt COx right* optimal mean arterial pressure calculated with right brain Cox, *LLA*

COx left lower limit of autoregulation calculated with left brain Cox, *LLA COx right* lower limit of autoregulation calculated with right brain Cox, *ULA COx left* upper limit of autoregulation calculated with left brain Cox, *ULA COx right* upper limit of autoregulation calculated with right brain Cox, *CO* cardiac output, *BMI* body mass index, *DM* diabetes mellitus, *SD* standard deviation, *ICU* intensive care unit, *IQR* interquartile range, *EuroSCORE* European System for Cardiac Operative Risk Evaluation

Table S2 The association between the tested parameters and heart failure (HF) outcome

**Parameters**		**non-HF group (*n* = 470)**	**HF group (*n* = 85)**	**MW test (*P*)**	**Unadjusted**		**Adjusted**	
					P	***OR***	***P***	***OR***
**LLA**	LLA COx left AUC (mmHg)	7.4 (95%CI 6.7 – 8.1)	9.6 (95%CI 7.4 – 11.8)	0.077	0.024	1.032	0.024	1.035
	LLA COx left percentage (%)	23.9 (95%CI 22.4 – 25.5)	25.9 (95%CI 22.1 – 29.6)	0.336	0.361	1.914	0.320	2.190
	LLA COx right AUC (mmHg)	7.7 (95%CI 7.0 – 8.4)	8.9 (95%CI 6.8 – 11.0)	0.217	0.224	1.017	0.208	1.019
	LLA COx right percentage (%)	24.8 (95%CI 23.2 – 26.3)	25.7 (95%CI 21.7 – 28.9)	0.683	0.796	1.207	0.668	1.375
**ULA**	ULA COx left AUC (mmHg)	12.4 (95%CI 11.4 – 13.3)	11.1 (95%CI 9.0 – 13.2)	0.230	0.305	0.986	0.266	0.984
	ULA COx left percentage (%)	29.9 (95%CI 28.4 – 31.5)	25.2 (95%CI 21.7 – 28.5)	0.015	0.019	0.147	0.240	0.141
	ULA COx right AUC (mmHg)	12.4 (95%CI 11.5 – 13.3)	11.8 (95%CI 9.3 – 13.7)	0.328	0.453	0.990	0.433	0.989
	ULA COx right percentage (%)	29.9 (95%CI 28.3 – 31.4)	25.7 (95%CI 22.7 – 28.8)	0.065	0.041	0.192	0.081	0.223
**MAPopt**	MAPopt COx left AUC (mmHg)	17.2 (95%CI 16.1 – 18.2)	24.5 (95%CI 20.1 – 29.0)	< 0.001	< 0.001	1.036	< 0.001	1.042
	MAPopt COx left percentage (%)	44.9 ± 28.2	51.1 ± 27.3	0.003	0.003	9.631	0.002	12.590
	MAPopt COx right AUC (mmHg)	17.5 (95%CI 16.4 – 18.5)	23.2 (95%CI 19.3 – 27.0)	0.002	0.001	1.031	0.001	1.034
	MAPopt COx right percentage (%)	45.8 ± 16.9	50.3 ± 2.2	0.031	0.028	5.354	0.026	6.459
**rSO_2 (%)**	rSO_2 left	71.5 ± 5.0	69.7 ± 5.5	0.001	0.003	0.932	0.016	0.941
	rSO_2 right	71.8 ± 5.0	70.5 ±5.1	0.018	0.017	0.947	0.026	0.944

**Other**	HR (beats/min)	64.2 ± 8.4	67.4 ± 13.0	0.167	0.004	1.035	0.020	1.032
	PPV (%)	13.6 ± 4.9	17.3 ± 9.8	0.001	< 0.001	1.079	< 0.001	1.071
	HRV	1.6 ± 1.7	3.0 ± 3.9	< 0.001	< 0.001	1.218	0.001	1.171
	LVEF (%)	58.0 ± 6.5	51.9 ± 9.0	< 0.001	< 0.001	0.906	< 0.001	0.919
	MAP SD	13.1 ± 3.3	13.9 ± 3.5	0.089	0.046	1.070	0.087	1.065
	CO (L/min)	5.0 ± 1.3	5.0 ± 1.5	0.678	0.997	1.000	0.469	1.074
**Correlation coefficient with CO**	MAPopt COx left and CO	-0.6 ± 0.3	-0.6 ± 0.3	0.612	0.477	0.728	0.597	0.976
	MAPopt COx right and CO	-0.6 ± 0.3	-0.7 ± 0.3	0.262	0.245	0.607	0.692	0.827
	LLA COx left and CO	-0.6 ± 0.3	-0.6 ± 0.3	0.233	0.217	0.582	0.629	0.794
	LLA COx right and CO	-0.6 ± 0.3	-0.7 ± 0.3	0.185	0.162	0.539	0.576	0.759
	ULA COx left and CO	0.6 ± 0.3	0.6 ± 0.3	0.816	0.612	1.231	0.899	0.943
	ULA COx right and CO	0.6 ± 0.3	0.6 ± 0.3	0.587	0.490	1.328	0.960	0.977
**Correlation coefficient with SV**	MAPopt COx left and SV	-0.6 ± 0.3	0.6 ± 0.3	0.600	0.341	0.674	0.618	0.796
	MAPopt COx right and SV	-0.6 ± 0.3	-0.6 ± 0.3	0.314	0.212	0.591	0.451	0.702
	LLA COx left and SV	-0.6 ± 0.3	-0.6 ± 0.3	0.241	0.196	0.570	0.414	0.678
	LLA COx right and SV	-0.6 ± 0.3	-0.6 ± 0.3	0.247	0.196	0.579	0.461	0.705
	ULA COx left and SV	0.6 ± 0.3	0.6 ± 0.3	0.797	0.453	1.361	0.708	1.187

ULA COx right and SV	0.6 ± 0.3	0.6 ± 0.3	0.543	0.385	1.427	0.728	1.172

Mean ± SD is used for parameter testing of normal distribution, and 95%CI is applied for non-parametric testing of non-normal distribution.

OR odds ratio, *LLA COx left AUC* area under curve of MAP below the lower limit of autoregulation calculated with left brain cerebral oximetry index, *LLA COx left percentage* time percentage of MAP below LLA based on left brain, *ULA COx left AUC* area under curve of MAP above ULA based on left brain, *ULA COx left percentage* time percentage of MAP above ULA based on left brain, *MAPopt COx left AUC* area under curve of MAP below MAPopt based on left brain, *MAP* mean artery pressure, *rSO_2 left* regional cerebral oxygen saturation of left brain, *rSO_2 right* regional cerebral oxygen saturation of right brain, *HR* heart rate, *PPV* pulse pressure variation, *HRV* heart rate variability, *LVEF* left ventricular ejection fraction, *MAP SD* standard deviation of mean arterial pressure, *CO* cardiac output, *MAPopt COx left and CO* correlation coefficient between MAPopt COx left and cardiac output, *LLA COx right and SV* correlation coefficient between LLA COx right and stroke volume, *MAPopt* optimal mean arterial pressure, *rSO_2* regional cerebral oxygen saturation, *LLA COx right AUC* area under curve of MAP below the lower limit of autoregulation calculated with right brain cerebral oximetry index, *LLA COx right percentage* time percentage of MAP below LLA based on right brain, *ULA COx right AUC* area under curve of MAP above ULA based on right brain, *ULA COx right percentage* time percentage of MAP above ULA based on right brain, *MAPopt COx right AUC* area under curve of MAP below MAPopt based on right brain, *MAPopt COx right percentage* time percentage of MAP below MAPopt based on right brain, *MAPopt COx right and CO* correlation coefficient between MAPopt COx right and cardiac output, *LLA COx left and CO* correlation coefficient between LLA COx left and cardiac output, *LLA COx right and CO* correlation coefficient between LLA COx right and cardiac output, *ULA COx left and CO* correlation coefficient between ULA COx left and cardiac output, *ULA COx right and CO* correlation coefficient between ULA COx right and cardiac output, *MAPopt COx left and SV* correlation coefficient between MAPopt COx left and stroke volume, *MAPopt COx right and SV* correlation coefficient between MAPopt COx right and stroke volume, *LLA COx left and SV* correlation coefficient between LLA COx left and stroke volume, *LLA COx right and SV* correlation coefficient between LLA COx right and stroke volume, *ULA COx left and SV* correlation coefficient between ULA COx left and stroke volume, *ULA COx right and SV* correlation coefficient between ULA COx right and stroke volume

References

1. Brady KM, Mytar JO, Lee JK, Cameron DE, Vricella LA, Thompson WR, et al. Monitoring cerebral blood flow pressure autoregulation in pediatric patients during cardiac surgery. Stroke. 2010;41(9):1957-62.
2. Liu X, Maurits NM, Aries MJH, Czosnyka M, Ercole A, Donnelly J, et al. Monitoring of optimal cerebral perfusion pressure in traumatic brain injured patients using a multi-window weighting algorithm. J Neurotrauma. 2017;34(22):3081-8.
3. Aries MJ, Czosnyka M, Budohoski KP, Kolias AG, Radolovich DK, Lavinio A, et al. Continuous monitoring of cerebrovascular reactivity using pulse waveform of intracranial pressure. Neurocrit Care. 2012;17(1):67-76.
4. Donnelly J, Czosnyka M, Adams H, Robba C, Steiner LA, Cardim D, et al. Individualizing thresholds of cerebral perfusion pressure using estimated limits of autoregulation. Crit Care Med. 2017;45(9):1464-71.
5. Liu X, Akiyoshi K, Nakano M, Brady K, Bush B, Nadkarni R, et al. Determining thresholds for three indices of autoregulation to identify the lower limit of autoregulation during cardiac surgery. Crit Care Med. 2021;49(4):650-60.
6. Brown CHt, Neufeld KJ, Tian J, Probert J, LaFlam A, Max L, et al. Effect of targeting mean arterial pressure during cardiopulmonary bypass by monitoring cerebral autoregulation on postsurgical delirium among older patients: a nested randomized clinical trial. JAMA Surg. 2019;154(9):819-26.
7. Ono M, Joshi B, Brady K, Easley RB, Zheng Y, Brown C, et al. Risks for impaired cerebral autoregulation during cardiopulmonary bypass and postoperative stroke. Br J Anaesth. 2012;109(3):391-8.
8. McDonagh TA, Metra M, Adamo M, Gardner RS, Baumbach A, Böhm M, et al. 2021 ESC Guidelines for the diagnosis and treatment of acute and chronic heart failure. Eur Heart J. 2021;42(36):3599-726.
